# Centering community-based maternal and child nutrition services in Bangladesh’s rural primary healthcare: what has potential to scale

**DOI:** 10.3389/fpubh.2025.1464792

**Published:** 2025-01-29

**Authors:** Safina Abdulloeva, Arti Bhanot, Mohd. Aziz Khan, Md. Mofijul Islam Bulbul, Mijanur Rahman, Kaosar Afsana, Thomas Forissier, Deepika Sharma, Abul Bashar Mohammad Khurshid Alam

**Affiliations:** ^1^UNICEF, Dhaka, Bangladesh; ^2^FHI 360, Durham, NC, United States; ^3^National Nutrition Services, Directorate General of Health Services, Dhaka, Bangladesh; ^4^James P Grant School of Public Health, BRAC University, Dhaka, Bangladesh; ^5^Directorate General of Health Services, Dhaka, Bangladesh

**Keywords:** community health workers, maternal and child nutrition, growth monitoring and promotion, social and behavior change, diets

## Abstract

**Introduction:**

The extensive network of community health workers in rural Bangladesh has the potential to deliver maternal and child nutrition services, while promoting linkages with healthcare facilities. A strategy for strengthening community-based nutrition services was developed and tested.

**Methods:**

The three-phased strategy included review of existing community-based systems, co-designing service package with multi-sector government representatives, and testing implementation feasibility. Integrated health and nutrition service delivery, supportive supervision, and increased accountability of local government were core components of the service package being implemented in selected geographies since March 2023. The assessment followed a mixed-method design with household survey of 1,166 pregnant women, mothers of children under-6 months and 6–23 months, and observations of 965 service delivery points along with qualitative study.

**Results:**

A higher proportion of children received growth monitoring and promotion (GMP) services through expanded program on immunization (EPI) sessions, with better compliance to service delivery protocol in intervention areas compared with controls. Maternal nutrition services of gestational weight gain monitoring and distribution of supplements were better available in intervention areas. However, minimum dietary diversity among pregnant women (69% intervention, 72% control), early initiation of breastfeeding (55% intervention, 51% control), and complementary feeding practices were comparable in intervention and control areas. Nutrition services were successfully integrated in supervision which earlier covered EPI and family planning. The local government contributed to strengthening nutrition services but at a small scale.

**Discussion:**

There is potential to scale-up GMP services through EPI and merging antenatal clinics with GMP and EPI such that all maternal and childcare services are available at the same place and same time. A coordinated investment and oversight from multiple national government departments is needed. At district and sub-district levels, scale-up requires joint annual planning of nutrition and EPI services, strengthened management of nutrition services, bridging health worker vacancies, introducing volunteers in sites with high EPI case load, capacity building, and supportive supervision. Replacement of multiple health and nutrition records with a single mother and child health and nutrition card is also feasible. However, behavior change interventions through home visits and courtyard meetings need more testing before recommending scale-up.

## 1 Introduction

Bangladesh has made significant progress in achieving global health and nutrition targets over the last decade and is on track to achieve the World Health Assembly 2025 target of a 40% reduction in the number of stunted children (1–3). However, some challenges persist. Repeat antenatal care (ANC) visits to maintain continuity of maternal nutrition services are missed by half the pregnant women who initiate these visits, and 35% still opt for home delivery (4). The prevalence of low birth weight is estimated at 23%, with over 70% of the effect explained by intrauterine growth retardation mainly due to poor maternal nutrition (5, 6). Nearly 60% of infants are not breastfed immediately after birth, and a similar proportion are not exclusively breastfed. The prevalence of child wasting increased between the two recent rounds of Demographic Health Survey from 8 to 11% (2, 4). Children 6–23 months fed minimum acceptable diet decreased in the same time frame from 35 to 29% (2, 4).

In 2011, the National Nutrition Services was launched to anchor nutrition programming and integrate activities into existing health and family planning programs. Nutrition services were to be embedded into routine primary healthcare (PHC) with gradual skill enhancement of community health workers (CHWs). In rural Bangladesh, there are three types of government supported CHWs—Community Health Care Providers (CHCP), Family Welfare Assistant (FWA), and Health Assistant (HA). The CHCP and HA are under the Directorate General of Health Services (DGHS) and FWA under the Directorate General of Family Planning (DGFP). The CHCP is stationed at the most accessible PHC structure for rural communities, the community clinic. The FWA and HA have outreach responsibilities in addition to seeing community clinic clients 2 and 3 days per week, respectively. A skilled Family Welfare Visitor, stationed at a union-level facility that serves the population of three or more community clinics, conducts outreach activities four to eight times per month.

The Government of Bangladesh in its 4th Health Nutrition and Population Sector Program (2016–2024) further intensified efforts to strengthen outreach services by CHWs (7, 8). Since 2016, a comprehensive competency-based training on nutrition, ranging from 3 days for CHWs to 5 days for their supervisors, was phased in to cover the entire country. The nutrition program currently focuses on strengthening last mile delivery of maternal infant and young child nutrition (MIYCN) services through capacity building of CHWs and their supervisors, increased investment in social and behavior change (SBC), and creating community demand for MIYCN services. It aims to integrate growth monitoring and promotion (GMP) into the widely accessible Expanded Program on Immunization (EPI) (9). Recognizing the inter-connectedness of health and nutrition services with other development sectors such as agriculture, education, and local governance, over 20 ministries collaborate under the Bangladesh National Nutrition Council (10). Under the Ministry of Local Government, Rural Development and Co-operatives (MOLGRD&C) elected representatives at the union level, the smallest rural administrative unit, form a Union Parishad that leads planning and implementation of several centrally funded development and welfare schemes (11).

To maximize the nutrition integration through community engagement, National Nutrition Services with other operational plans (thematic verticals under Health Population and Nutrition Sector Program), United Nations Children’s Fund (UNICEF), and FHI 360 designed and tested an innovative strategy for community outreach, engagement, and accountability for improving MIYCN and linked PHC services in rural Bangladesh (referred to as community-based engagement strategy). The theoretical strength of delivering MIYCN integrated with PHC platforms is recognized at the policy level (9). However, the practical aspects including feasibility, implementation challenges, and sustainability of integrating MIYCN into PHC are not fully understood in Bangladesh context. Thus, an independent assessment was conducted in addition to routine system monitoring to generate evidence on feasibility and effectiveness of implementing the community-based services package. The objectives of the assessment were to (1) assess effect on GMP and maternal nutrition service coverage along with system readiness and compliance to service protocols, (2) impact of the intervention on the infant and young child feeding practices and dietary diversity among pregnant women, and (3) capture the factors influencing implementation and pre-requisites for possible scale-up in future.

## 2 Methods

### 2.1 Community-based engagement strategy

The strategy consisted of three phases: phase (1) review and mapping of community-based health and nutrition initiatives in rural Bangladesh (2020–2021), phase (2) consultative development of a community-based service package (2021–2022), and phase (3) implementation of this service package in 12 upazilas since 2023, including system preparedness activities to initiate implementation. On average, each upazila includes 10–11 unions although this number can vary significantly. It ranges from 5 to 16 in the 12 implementation upazilas.

In phase 2, the service package co-designed with government leadership, first line supervisors, and CHWs consisted of three components: (1) integrated community-based services through home visits, courtyard meetings, EPI outreach, and satellite clinics, (2) supportive supervision balancing administration, capacity building, and problem-solving, and (3) institutionalized accountability for MIYCN and inter-linked services by community groups and Union Parishads. Table 1 summarizes the routine services, that is, services already available under PHC and augmented services as per community-based engagement strategy.

**Table 1:** Routine services versus augmented services under community-based engagement strategy.

Service component	Routine services	Augmented services
Component 1: Integrated community-based services	GMP at community clinics only. GMP includes measuring MUAC, weight, and height, updating growth charts, providing counseling, and making referrals for children suspected of being affected by SAM.	GMP at all EPI outreach sites (24 in every union) every month following standard protocol (height measured at community clinics only). HA issues referral slip to all children suspected of growth faltering, advising caregiver to visit the community clinic for further screening of the child.
	Satellite clinics* ranging from 4 to 8 every month depending on Family Welfare Visitor’s availability.	All satellite clinics co-located with GMP and EPI sites to create a one-stop service for maternal and childcare.
	Maternal nutrition services available at satellite clinics. However, satellite clinics not organized in absence of Family Welfare Visitor (skilled worker).	Maternal nutrition services of iron folic acid and calcium tablets distribution, weight gain monitoring and counseling delivered by FWA (a CHW) even in absence of Family Welfare Visitor. Introduction of maternal nutrition protocol for ANC.
	FWA undertakes home visits for registration of pregnant women, at least one visit post-partum to check on mother-baby preferably within 24 h in addition to visits to eligible couples for family planning services.	FWA undertakes at least two home visits to pregnant women, in first trimester to register and promote uptake of ANC services and third trimester to confirm continuity of ANC and preparation for institutional delivery. Trained and equipped with maternal nutrition counseling resources.
	FWAs organize one courtyard meeting (group counseling) per month.	FWAs organize at least one courtyard meeting on a pre-decided theme. Trained on group counseling for (1) maternal nutrition in ANC, PNC, (2) GMP and infant and young child feeding, and (3) early childhood care and development.
	HA undertakes home visits before EPI Day to inform families and promote attendance.	HA trained on GMP and infant and young child feeding to counsel mother/family members during these visits.
	Families maintain separate records for ANC, immunization, GMP.	Mother and child health and nutrition card covering all records from conception till child is 5 years of age, along with messages on recommended health and nutrition practices, provided to all pregnant women on registration and to mothers if they missed it during pregnancy. CHWs and other service providers trained on using the card.
Component 2: Supportive supervision	Supervision visits checklists cover EPI and family planning services. Targets driven supervision. Upazila monthly review meetings cover EPI and family planning as main agenda items.	Checklists cover nutrition services (observations at GMP sessions, satellite clinics, home visits, and courtyard meetings) in addition to EPI and family planning. Microsoft Excel spreadsheet introduced to consolidate and analyze supervision data. Realistic supervision visits targets set to meet all job expectations. Supervisor trained on observing services, providing feedback, and on the job training. Upazila review meetings include nutrition services in agenda.
Component 3: Institutionalizing accountability through community groups and Union Parishads	Community groups are involved in management of community clinics and promote increased utilization of services. CHCP organizes monthly meetings with group members. Union Parishad ideally develops annual plans with budget for health and development activities, holds review meetings with representative from different departments and organizes public meetings.	Members of community groups and Union Parishad oriented to augmented service package and potential of their support. Union Parishads guided on activities that can be funded by them (examples-volunteers for GMP sites, SBC resources, logistics for courtyard meetings).

*Outreach clinics for ANC, PNC, family planning services.

The augmented services were delivered entirely by government supported service providers, supervisors, and managers. One coordinator, appointed through UNICEF-FHI 360, was stationed at the upazila level to provide technical assistance at both managerial and supervisory levels. S/he conducted field visits with and without system supervisors and facilitated the information needs for the monthly upazila review meetings of DGFP and DGHS by supporting system statisticians in data consolidation and analysis.

In phase 3, the selection of 12 upazilas was based on relatively poorer performance on selected nutrition indicators compared to other upazilas in the same district, completion of comprehensive competency-based training on nutrition for all CHWs and their supervisors and consultation with local and national government. The upazilas were Ajmeriganj and Baniachong in Habiganj district, Begumganj and Subarnachar in Noakhali district, Bhola Sadar and Daulatkhan in Bhola district, Durgapur and Purbadhala in Netrokona district, Kaliganj and Shyamnagar in Satkhira district, and Nageshwari and Ulipur in Kurigram district. Preparedness for implementation was achieved through orientations from national to upazila levels, 1-day training on augmented service package for CHWs and 2-day training for supervisors, and the procurement and deployment of anthropometric equipment including newborn, child and adult weighing scales, length and height boards measuring up to 210 cm, and mid-upper arm circumference (MUAC) tape for children along with counseling resources. At the upazila level, the annual EPI microplans which outline the number of EPI eligible children under-2 years of age, along with a calendar of EPI sessions, were modified to include GMP sessions, satellite clinics, and courtyard meetings.

### 2.2 Assessment design and sampling

The assessment was a mixed-method study conducted by BRAC James P Grant School of Public Health. The upazilas with at least 9 months of package implementation were considered for the assessment to ensure full period of ANC coverage for near-term pregnant and recently delivered women. 6 of the 12 upazilas met this criteria. Of these six, two namely Begumganj from district Noakhali and Kaliganj from district Satkhira were selected for the assessment based on relatively fewer hard-to-reach areas and mid-to-large area and population. For the quantitative study, a control upazila, that is, where augmented service package was not implemented, was selected from the same district but not having common borders with the implementation upazila (also referred to as intervention upazila). Controls were matched against demographic variables including head count ratio for people living below poverty line, access to electricity and housing condition, prevalence of child stunting and underweight, and coverage of selected health and nutrition services.

#### 2.2.1 Qualitative study

The qualitative study was undertaken at two time points in the intervention upazilas: Round 1 (May and June 2023) early in the implementation as an exploratory study and Round 2 (December 2023 and January 2024) late in the implementation to provide deeper insights into the implementation processes and better understanding of quantitative findings. Selection criteria for participants of key informant interviews (KIIs) were minimum posting of 3 months and only those CHWs and supervisors who were trained on augmented service package in early 2023, or received refresher training, were considered for in-depth interview (IDI) (Table 2).

**Table 2:** Qualitative study participants, sample size, and tools used.

Participants/Observations	Round 1	Round 2	Tools used
National level managers	5	5	KII
District and upazila managers	17	21	KII
Frontline supervisors	14	16	IDI
CHWs	25	32	IDI
Service delivery observations	19	21	Observation checklist
Total	**80**	**95**	

Bold values indicate total sample size for qualitative and quantitative study.

#### 2.2.2 Quantitative study

The quantitative study was a one-time household survey covering 1,166 population with 337 pregnant women, 490 children under-6 months, and 339 children 6–23 months. The sample size calculation for pregnant women was based on iron folic acid (IFA) consumption for at least 90 days, infants under-6 months on exclusive breastfeeding, and children 6–23 months on minimum acceptable diet (2, 12) (Table 3).

**Table 3:** Sample size estimation for target groups.

Target group (indicator used)	Pre-intervention coverage (4)	Post-intervention expected coverage	DE	Non-response	2 arms [4 upazilas]	Per upazila
Pregnant women 15–45 years (IFA consumption)	52%	72%	1.7	5%	326	81
Children <6 months (exclusive breastfeeding)	65%	80%	1.7	5%	488	122
Children 6–23 months (minimum acceptable diet)	52%	72%	1.7	5%	326	81
Total					**1,140**	**284**

Bold values indicate the total estimated sample for the target groups and observations, which was lower than actual covered.

Pregnant women (15–45 years) who were residents of the area and/or living in that area for the 12 months preceding the survey were considered. Married adolescent mothers were included, and ethical procedures for minors as respondents were followed. Six unions were randomly selected from each upazila. List of pregnant women and children were obtained from CHWs and verified against the list at the community clinics in these six unions. Randomization was done individually for each sampling frame of participants (i.e., pregnant women, children under-6 months, and children 6–23 months) stratified by upazila. A random number generator was used to select the relevant sample size per upazila, and participant names were compiled into a participant list on Microsoft Excel and finalized for each upazila.

Service delivery observations were made at 965 sites covering 207 service providers. Sample size calculations for observation of ANC checkups and GMP sessions were based on estimates of service utilization (12, 13) (Table 4).

**Table 4:** Sample size estimation for observations.

	Pre-intervention coverage (4)	Post-intervention expected coverage	DE	Non-response	2 arms [4 upazilas]	Per upazila
a. System readiness (training, tools, equipment)	50%	75%	1.7	5%	205	51
b. Protocol compliance						
Observation of ANC checkup (nutrition)	30%	50%	1.7	5%	254	64
Observation of GMP	35%	50%	1.7	5%	600	150
Total					**854**	**214**

Bold values indicate the total estimated sample for the target groups and observations, which was lower than actual covered.

Observation visits were planned to ensure maximum coverage of all unions within each upazila. List of CHWs available with upazila coordinators were used to randomly select the three types of CHWs (CHCPs, FWAs, and HAs). Once CHW list was generated, annual EPI schedules available in upazila level microplans were used to select GMP sessions for observation visits. Similarly, monthly satellite clinic schedules available in the same microplan were used to select sites for ANC checkup observations for all four upazilas.

### 2.3 Data collection, tools, and training

#### 2.3.1 Qualitative study

The KIIs and IDIs were conducted using detailed guides covering background information, respondent’s role in service delivery, use of job aids, system readiness to support delivery of services, collection and use of data, and perspectives on improving service delivery. Interview time and location were decided in consultation with respondents. National level interviews were undertaken by senior research team and field interviews by trained enumerators supported by a research assistant.

Data enumerators for qualitative study underwent 7-day training. Training included overall health system structure, objectives of the study, ethical issues and safeguarding, use of interview guides, taking informed consent, sampling strategy, respondent selection and how to conduct interviews, note-taking, and transcript writing.

#### 2.3.2 Quantitative study

Indicators to assess effectiveness of the package were Minimum Dietary Diversity-Women (MDD-W), early initiation of breastfeeding and exclusive breastfeeding among children under-6 months and MDD, and minimum meal frequency and minimum acceptable diet for children 6–23 months (14, 15). As data on maternal diet were unavailable from large-scale surveys, IFA consumption was used as proxy to estimate sample size for pregnant women as described under “Assessment design and sampling.” Among children 6–23 months, unhealthy food consumption following WHO definition was also estimated (15).

Three quantitative closed-ended questionnaires were developed for pregnant women, and mothers of children under-6 months and 6–23 months. Observation checklist for GMP and maternal nutrition services at ANC were based on protocols introduced under community-based engagement package. Both checklists covered availability of logistics, steps in service delivery, and recording client information. All questionnaires were developed digitally using Kobo Toolbox, and data were collected via the Kobo Collect application v2023.2.4. Data enumerators were provided with electronic tablets pre-installed with Kobo Collect.

### 2.4 Data analysis

#### 2.4.1 Qualitative study

Audio recording was done with consent in addition to capturing field notes. Audio recordings of the KIIs and IDIs were transcribed verbatim in Bangla by research assistants. Transcribed data were stored in encrypted servers and de-identified before analysis. ATLAS.ti 9 software was utilized for coding of the transcriptions. Thematic analysis was conducted using the inductive coding method. A codebook was generated after coding was completed for all the transcriptions. Data were reviewed and analyzed using the generated codebook.

#### 2.4.2 Quantitative study

Stata 14 was used for quantitative analysis. Weights were used to report the aggregated estimate of coverage of the services after adjusting for the population size of the upazilas. Sample characteristics and summary statistics were reported between the intervention and the control upazilas. Sub-categories were created to compare background characteristics including wealth index. The wealth index was constructed using household asset data via principal components analysis as used in Demographic Health Surveys. Households were assigned a score based on the ownership of various assets, access to utilities such as electricity, sanitation, and housing characteristics such as flooring materials, and the number of rooms. Based on score rankings, households were divided into five equal groups representing relative wealth categories, from the poorest to the richest.

Binary variables were summarized as proportions, and continuous variables were summarized as mean values with standard deviations. The chi-square (*χ*2) test was used to test for significant differences between categorical variables and *t*-test for continuous variables in the study groups.

### 2.5 Ethics approval

The assessment was approved by the ethical committee of the Institutional Review Boards of James P Grant School of Public Health and FHI 360 Protection of Human Subjects Committee. The consent forms and tools were designed in English, translated into Bangla, verified, and approved by the ethical review committees. Both parental consent and minor assent forms were used adolescent girls (less than 18 years) and their guardians. A copy of signed consent forms, along with the name, phone number, and address of the study coordinator, was offered to every study participant by the data collectors.

### 2.6 System monitoring

The monitoring framework for community-based engagement was based on information available through the management information system (MIS) of the government. A total of 15 service delivery indicators for which data were routinely collected by CHWs, compiled by their supervisors and ultimately available with the upazila managers, were the mainstay of monitoring. Simple manual modifications such as adding columns for anthropometric measurements were made in the existing reporting formats to ensure reporting on GMP services from EPI outreach sites which was earlier unavailable through MIS. Data for children suspected of growth faltering were highlighted in these formats for follow-up. Monitoring data were analyzed using Microsoft Excel.

## 3 Results

### 3.1 Background characteristics of household survey participants

Among the sampled pregnant women, the intervention and control upazilas in Noakhali and Satkhira were matched on eight and four of the nine background characteristics, respectively (Table 5). Among mothers of children under-6 months, intervention and control upazilas matched on four and five of the seven characteristics in Noakhali and Satkhira, respectively, while among mothers of children aged 6–23 they matched on six characteristics in Noakhali but only two in Satkhira (Table 6).

**Table 5:** Background characteristics of pregnant women.

Characteristics	Satkhira			Noakhali		
	Intervention	Control	*p*-value	Intervention	Control	*p*-value
	Kaliganj	Tala		Begumganj	Chatkhil	
	*N* = 82	*N* = 90		*N* = 81	*N* = 84	
Number of household members (Mean ± SE)	4.6 ± 0.2	4.5 ± 0.2	0.565	5.7 ± 0.3	5.3 ± 0.3	0.266
Per capita household expenditure in previous week (Mean ± SE)	717.4 ± 35.3	469.8 ± 25.4	<0.001*	814.3 ± 94.0	959.9 ± 57.5	0.185
Age of pregnant women (%)						
<20 years	25.6	21.1	0.619	24.7	17.9	0.009*
20 to 25 years	24.4	33.3		40.7	25.0	
26 to 30 years	29.3	27.8		24.7	28.6	
>30 years	20.7	17.8		9.9	28.6	
Multiparous (%)	28.1	41.1	0.080	45.7	31.0	0.056
Gestational age (%)						
First trimester	17.1	34.4	0.024*	21.0	20.2	0.970
Second trimester	62.2	53.3		56.8	56.0	
Third trimester	20.7	12.2		22.2	23.8	
Religion (%)						
Islam	96.3	77.8	<0.001*	100.0	96.4	0.246
Hindu	3.7	22.2		0.0	3.6	
Occupation of household head (%)						
Business/Trader	34.2	57.8	<0.001*	25.9	31.0	0.144
Service/Salaried Worker	22.0	26.7		48.2	33.3	
Unskilled/Housewife/Unemployed	43.9	15.6		25.9	35.7	
Years of formal education (%)						
<= 5 years	18.3	14.4	0.350	7.4	7.1	0.874
> 5 to <=10 years	65.9	61.1		69.1	72.6	
> 10 years	15.9	24.4		23.5	20.2	
Wealth index (%)						
Poorest	23.2	17.8	0.006*	30.9	15.5	0.182
Poorer	29.3	11.1		17.3	19.1	
Middle	20.7	20.0		14.8	22.6	
Richer	14.6	24.4		19.8	20.2	
Richest	12.2	26.7		17.3	22.6	

*Indicates significant change at *P* < 0.05.

**Table 6:** Background characteristics of mothers.

Characteristics	Children under-6 months						Children 6–23 months					
	Satkhira			Noakhali			Satkhira			Noakhali		
	Intervention	Control	*p*-value	Intervention	Control	*p*-value	Intervention	Control	*p*-value	Intervention	Control	*p*-value
	Kaliganj	Tala		Begumganj	Chatkhil		Kaliganj	Tala		Begumganj	Chatkhil	
	*N* = 120	*N* = 124		*N* = 122	*N* = 124		*N* = 82	*N* = 90		*N* = 81	*N* = 84	
Number of household member (Mean ± SE)	5.6 ± 0.2	5.3 ± 0.1	0.206	6.2 ± 0.2	6.1 ± 0.2	0.759	5.5 ± 0.2	4.9 ± 0.2	0.040*	6.1 ± 0.3	6.0 ± 0.3	NS
Per capita household expenditure in previous week (Mean ± SE)	575.0 ± 22.4	381.7 ± 20.1	<0.001*	719.8 ± 48.2	963.3 ± 54.0	<0.001*	624.39 ± 31.83	426.56 ± 32.17	<0.001*	783.95 ± 74.70	946.86 ± 64.52	NS
Age of mother (%)												
<20 years	21.7	22.6	0.562	18.0	16.9	0.872	28.2	23.5	NS	11.1	19.3	NS
20 to 25 years	33.3	31.5		38.5	40.3		36.5	29.6		40.0	26.5	
26 to 30 years	20.0	14.5		23.8	20.2		16.5	30.9		18.9	27.7	
>30 years	25.0	31.5		19.7	22.6		18.8	16.1		30.0	26.5	
Religion (%)												
Islam	92.5	87.1	0.206	97.5	99.2	0.368	92.9	77.8	0.007*	93.3	96.4	NS
Hindu	7.5	12.9		2.5	0.8		7.1	22.2		6.7	3.6	
Occupation of household head (%)												
Business/Trader	53.3	61.3	0.013*	21.3	32.3	<0.001*	44.7	49.4	0.002*	30.0	38.6	0.010*
Service/Salaried Worker	20.8	27.4		61.5	33.1		24.7	40.7		53.3	31.3	
Unskilled/Housewife/Unemployed	25.8	11.3		17.2	34.7		30.6	9.9		16.7	30.1	
Years of formal education (%)												
<= 5 years	10.8	8.9	0.465	7.4	4.8	0.705	5.9	8.6	NS	7.8	3.6	NS
> 5 to <=10 years	68.3	63.7		65.6	66.9		71.8	59.3		60.0	77.1	
> 10 years	20.8	27.4		27.1	28.2		22.9	32.1		32.2	19.3	
Wealth index (%)												
Poorest	23.7	18.6	0.477	30.6	10.5	<0.001*	30.6	9.9	<0.001*	26.1	16.9	NS
Poorer	20.0	20.2		20.7	20.2		23.5	16.1		13.6	24.1	
Middle	21.7	18.6		20.7	17.7		18.8	21.0		22.7	16.9	
Richer	21.7	18.6		12.4	27.4		18.8	21.0		17.1	22.9	
Richest	15.0	24.0		15.7	24.2		8.2	32.1		20.5	19.3	

^*^Indicates significant change at *P* < 0.05.

### 3.2 GMP for EPI eligible children

**Figure 1:**
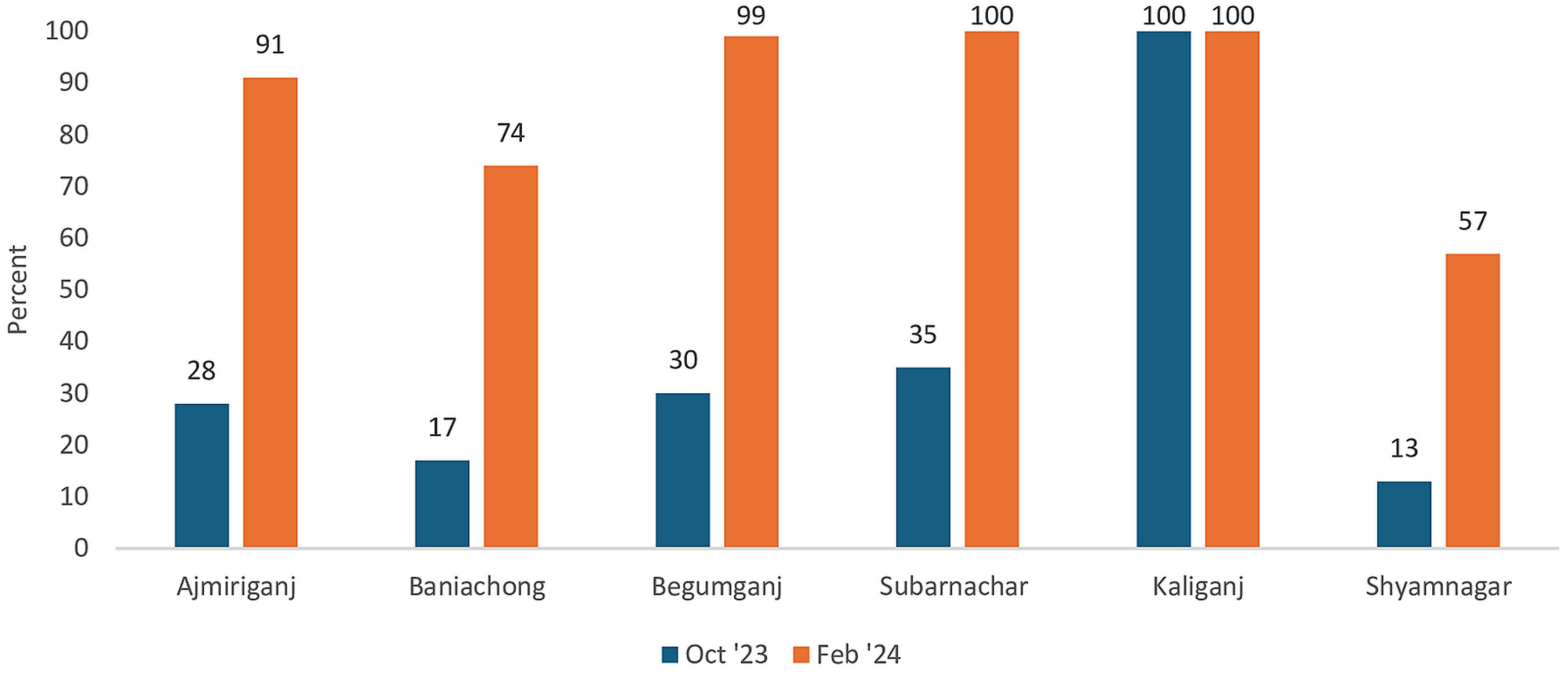
Coverage of child weight measurement across all intervention upazilas (monitoring data). Data for October 2023 and February 2024 were compared as all sites were equipped by October 2023 and one year of implementation was completed in February 2024. Coverage was over 100% in Kaliganj (139% in October 2023 and 124% in February 2024) and Subarnachar (108% in February 2024) due to some non-EPI eligible children also availing services and catchup due to disruption in organization of EPI session in January 2024, respectively.

Monitoring data supported the gradual uptick in reach of child weight measurement services across all intervention upazilas (Figure 1). On an average, 300 GMP-EPI sessions are held every month in Kaliganj and 380 in Begumganj. The number of children at each EPI session ranged from under 40 to almost 100.

Based on the household survey, awareness on GMP services was significantly higher among mothers of children under-6 months and 6–23 months in Kaliganj compared with control upazila (91% vs. 17%; 80% vs. 9% *p* < 0.001), so was receipt of counseling services (84% vs. 13%; 74% vs. 6% *p* < 0.001). The reported receipts of counseling services in Begumganj and control upazila were comparable at 20 and 25% respectively, for mothers of children 6–23 months (Data not available in tables). Higher proportion of children were weighed, had length/height and MUAC measured, followed by correct plotting and counseling in intervention upazilas than control based on observations of GMP sessions (Table 7).

**Table 7:** GMP services being provided per protocol based on observations.

GMP services per protocol (%)	Satkhira			Noakhali		
	Intervention	Control	*p*-value	Intervention	Control	*p*-value
	Kaliganj	Tala		Begumganj	Chatkhil	
	*N* = 153	*N* = 153		*N* = 209	*N* = 160	
Accurately recording children’s age	97.4	98.7	0.984	99.5	100.0	0.984
Taking children’s weight	97.4	5.2	<0.001*	88.0	1.9	<0.001*
Taking children’s length^#^	24.2	0	-	26.8	0	-
Taking children’s MUAC	45.8	5.2	<0.001*	23.4	0	-
Plotting the GMP measurements in color coded zones on GMP chart	94.1	0.7	<0.001*	66.5	0.7	<0.001*
Identifying children’s nutritional status according to color zones of GMP chart	91.5	0	-	64.6	0	-
Including information on GMP services in the tally list/sheet	78.4	0	-	32.1	0	-
Listing the non-attendees and follow-up	28.8	33.3	0.387	47.9	46.3	0.761
Advice on the nutrition of the child according to the growth status of the child as per GMP card	66.0	1.3	<0.001*	40.7	3.1	<0.001*
Using SBC materials	17.0	0	-	29.7	6.9	<0.001*

^#Available at community clinics only.^

Monitoring data on referral revealed not as many children were identified with growth faltering and referred as would be expected considering the national prevalence of underweight and wasting at 22 and 11%, respectively (4).

### 3.3 Maternal nutrition services

The monitoring data for ANC and maternal nutrition services were deemed unusable as it indicated 100% or higher coverage throughout the implementation period. Observations at satellite clinics revealed higher proportion of women were weighed, had blood pressure measured, underwent physical examination, and received calcium tablets in intervention upazilas compared with controls (Table 8). In the household survey, almost all pregnant women reported consuming IFA tablets in their current pregnancy in both intervention and control upazilas (93% vs. 98%). Observations revealed IFA tablets were provided more frequently in Begumganj compared to the control upazila, but service providers missed advising on dosage and side effects.

**Table 8:** Maternal nutrition and ANC services being provided per protocol based on observations.

Maternal nutrition and ANC services	Satkhira			Noakhali		
	Intervention	Control	*p*-value	Intervention	Control	*p*-value
	Kaliganj	Tala		Begumganj	Chatkhil	
	*N* = 73	*N* = 65		*N* = 87	*N* = 65	
Determining the number of ANC visit	89.0	90.8	0.737	92.0	81.5	0.055
Determining expected date of delivery	35.6	67.7	<0.001*	78.2	69.2	0.112
Height measurement	28.8	0	-	17.0	0	-
Weight measurement	78.1	41.5	<0.001*	57.5	10.8	<0.001*
Blood pressure measurement	86.3	40.0	<0.001*	36.8	9.2	<0.001*
Clinical examination for anemia	46.6	17.0	<0.001*	19.5	9.2	0.079
Confirming the blood and urine tests were done	32.9	10.8	0.002*	10.3	0	-
Registering into register book assigning an ID	30.1	64.6	<0.001*	72.4	67.7	0.558
Providing mother child health and nutrition card	34.3	0	-	57.5	0	-
Updating mother child health and nutrition card (among those who received)	83.6	0	-	66.7	0	-
Determining the health status of pregnant women according to the physical examinations	74.0	40.0	<0.001*	43.7	13.9	<0.001*
Providing IFA tablets	83.6	93.9	0.068	63.2	10.8	<0.001*
Providing Calcium tablets	82.2	67.7	0.048*	46.0	9.2	<0.001*
Advising on dosage, side effects and management for IFA and calcium tablets	22.0	53.9	<0.001*	26.4	0	-
Listing non-attendees and follow-up	23.3	39.1	0.046*	39.1	92.3	<0.001*
Providing nutritional counseling	71.2	76.9	0.447	83.9	53.9	<0.001*
Using job aids	30.1	0	-	43.7	0	-
Using SBC materials	30.1	4.6	<0.001*	38.0	6.2	<0.001*

^*^Indicates significant change at *P* < 0.05.

Despite being a longstanding component of FWA job description, home visits to recently delivered women and newborns within 48 h of birth, that is, double the recommended cutoff time, were very low based on household survey (Table 9). Home visit coverage, without a 48-h cutoff, was higher in the intervention upazilas according to monitoring data for February 2024 compared to the household survey, at 31% in Kaliganj (*N* = 313 deliveries) and 65% in Begumganj (*N* = 839 deliveries).

**Table 9:** Visits to recently delivered women and newborn based on household survey.

Indicators (%)	Satkhira			Noakhali		
	Intervention	Control	*p*-value	Intervention	Control	*p*-value
	Kaliganj	Tala		Begumganj	Chatkhil	
	*N* = 120	*N* = 124		*N* = 122	*N* = 124	
Visited by any service provider within 48 h. of most recent birth	3.3	2.4	0.719	1.6	6.5	0.102
Service provider supported them to breastfeed baby during any home visit	4.2	15.3	0.003*	23.1	40.3	0.004*
Service provider provided IFA tablets during any home visit	5.8	4.8	0.729	13.1	15.3	0.620

### 3.4 Behavior change

Findings were comparable across intervention and control upazilas on behavior change indicators of MDD-W, breastfeeding, and complementary feeding (Tables 10, 11).

**Table 10:** Food group consumption and MDD-W estimates for pregnant women, household survey.

Consumption of food groups (%)	Satkhira			Noakhali		
	Intervention	Control	*p*-value	Intervention	Control	*p*-value
	Kaliganj	Tala		Begumganj	Chatkhil	
	*N* = 82	*N* = 90		*N* = 81	*N* = 84	
Grains, roots, tubers, and plantains	100.0	98.9	1.000	98.8	100.0	1.000
Pulses	37.8	42.2	0.555	56.8	45.8	0.159
Nuts and seeds	26.8	28.9	0.764	22.5	15.7	0.266
Milk and milk products	56.1	76.7	0.004*	49.4	67.5	0.019*
Meat, fish, and poultry	89.0	80.0	0.104	85.2	81.0	0.469
Eggs	54.9	58.9	0.596	46.9	59.5	0.105
Dark green leafy vegetables	47.6	58.9	0.137	43.2	61.5	0.019*
Vitamin A-rich fruits	43.2	42.2	0.896	29.6	38.1	0.251
Other vegetables	54.9	58.9	0.596	46.9	51.2	0.583
Other fruits	42.7	64.4	0.004*	44.4	47.6	0.683
Consumed > = 5 food groups	73.2	74.4	0.849	65.4	69.1	0.621

**Table 11:** Infant and young child feeding estimates, household survey.

Breastfeeding practices (%) (Children < 6 months)	Satkhira			Noakhali		
	Intervention	Control	*p*-value	Intervention	Control	*p*-value
	Kaliganj	Tala		Begumganj	Chatkhil	
	*N* = 120	*N* = 124		*N* = 122	*N* = 124	
Ever breastfed	100.0	98.4	0.498	100.0	100.0	-
Initiation of breastfeeding within an hour of birth	52.5	57.3	0.455	45.9	45.2	0.907
Exclusive breastfeeding (fed only breast milk in the last 24 h)	52.5	51.6	0.890	59.0	62.1	0.621
Exclusively breastfed for the first 2 days after birth	50.8	43.6	0.254	29.5	29.8	0.955
Mixed milk feeding	19.2	20.2	0.845	23.8	13.7	0.043*
**Complementary feeding practices (%) (Children 6–23 months)**	*N* = 85	*N* = 81		*N* = 90	*N* = 83	
Minimum dietary diversity	55.3	60.5	0.498	52.2	49.4	0.710
Minimum meal frequency	89.4	87.7	0.722	75.6	71.1	0.506
Minimum acceptable diet	50.6	59.3	0.262	46.7	42.2	0.552
Eggs and/or flesh food consumption	81.2	92.6	0.030*	71.1	69.9	0.859
Sweet beverage consumption	22.4	38.3	0.025*	51.1	32.5	0.013*
Unhealthy food consumption	75.3	81.5	0.334	65.6	61.5	0.575
Zero vegetable or fruit consumption	30.6	33.3	0.705	31.1	38.6	0.304
Bottle feeding	22.4	16.3	0.322	36.7	30.1	0.362

*Indicates significant change at *P* < 0.05.

A significantly higher proportion of mothers reported being counseled on exclusive breastfeeding in Kaliganj than control (60% vs. 44%, *p* = 0.01), but the reverse was noted for Begumganj and control (50% vs. 69%, *p* = 0.002). Reported counseling on age-appropriate feeding (46% vs. 24%, *p* = 0.002) and responsive feeding (19% vs. 7% *p* = 0.03) was also higher in Kaliganj than control upazila. Counseling on early childhood care and development and play-based activities were less frequent. However, nurturing care and responsive feeding were discussed during courtyard meetings.

### 3.5 Feasibility of implementation

The findings from the qualitative component provided insights on feasibility of implementation.

#### 3.5.1 Availability of CHWs and volunteers

National and upazila level officials strongly recommended dedicated service providers for nutrition services in villages. CHW vacancy ranged from 20% to a just over 40% across implementation upazilas.

*“HR gap is a big issue. Previously ‘Pushthi Apa’ [female nutrition worker] worked at the field level… ‘Pushthi Apa’ would go from house to house making people aware of nutrition. We need someone focused only for nutrition service like her who will counsel and motivate people … if any volunteer or someone similar can be provided, that will work well.” (KII, National Level).*

*“In the absence of multi-purpose volunteers* [government supported volunteers piloted in few upazilas between 2019 and 2023]*, we are providing GMP services … There are a lot of vaccines and administering them consumes considerable time. Consequently, providing GMP services afterwards poses challenges, particularly in measuring weight and height, providing counselling, filling the cards. Consequently, our [HA] service quality deteriorated both in terms of quality and quantity.” (IDI, HA, Kaliganj, Satkhira).*

Union Parishads supported volunteers in some unions for promoting GMP and other MIYCN services. Selection of volunteers was prioritized in unions with high number of EPI eligible children. Such investment by Union Parishad was possible in upazilas where DGHS upazila managers actively engaged with the Upazila and Union Parishads and other officials of the MoLGRD&C. As Union Parishads are under a different Ministry, common platforms for discussion with health and family planning staff are limited. *There is a lack of communication with me and them [Union Parishad representative] I feel there is a great opportunity for the Union Parishad to play a major role. They need more involvement with us. If we want to make the program successful, we need to increase communication with all those who can influence it.—KII, Upazila manager, Begumganj, Noakhali.*

#### 3.5.2 Availability of equipment, protocols, and resources to promote nutrition services

The qualitative study revealed improved availability of anthropometric equipment, GMP protocols, and SBC resources between the two rounds of data collection. CHWs mentioned curiosity among mothers about the mother and child health and nutrition card and understanding the growth of their children.

*“Having the card has been very beneficial. In the card, we show them age-appropriate recommendations, type of foods they should feed and other images. Even if they forget, seeing the card they remember.” IDI, Begumganj, Noakhali.*

#### 3.5.3 Capacity building through trainings and supportive supervision

The 1-day community-based engagement training for CHWs was perceived inadequate by CHWs and their supervisors. Continuing field support and refresher trainings were suggested. Completion of the supportive supervision checklists and data reporting improved between rounds 1 and 2 of the qualitative study. Supervisors mentioned challenges in filling a lengthy checklist during round 1. The checklist was simplified based on this feedback, and by round 2, it was fully executed. However, supervisors were hesitant to report non-compliance to protocols CHWs as they feared it could reflect poorly on the CHWs’ performance.

#### 3.5.4 Joint inter-department annual planning and progress reviews

Most respondents acknowledged coordination challenges between DGFP and DGHS. They alluded to annual microplanning for nutrition services along with EPI and joint DGFP and DGHS review meetings at all administrative levels as good initiatives to improve coordination. However, there were concerns on sustainability of such platforms post-project life. Respondents from national and upazila level recommended continued UNICEF-FHI360 support for community-based engagement for longer period of time to have a notable impact on nutritional indicators.

## 4 Discussion

The assessment findings provided evidence in support of the different components of the community-based service package. First, evidence supported layering GMP services consisting of weighing, MUAC measurement, plotting growth chart, and counseling (referral), on the EPI platform. In addition, co-locating satellite clinics such that all preventive and promotive services for pregnant women, breastfeeding mothers, and children were available at the same place and same time was possible in the context of rural Bangladesh. Leveraging the high footfall and efficient service delivery of the EPI platform made it ideal for integrating other preventive services. While GMP services were earlier available at community clinics, they were underutilized as children were brought to community clinics only when unwell. The opportunity cost of visiting community clinic for only GMP services was prohibitive for most families that stayed at a distance from these clinics. The success of GMP integration with EPI defied known challenges of increased workload of CHWs due to high vacancies and low community demand for GMP (16, 17). Volunteers supported by Union Parishads helped in managing high EPI case load sites but were available in very few areas. Program managers and service providers acknowledged systemic challenges but also demonstrated a very positive and optimistic view regarding integrating GMP into EPI. As integration of GMP in EPI with additional human resource support was also recommended in the mid-term review of the ongoing health, population, and nutrition sector program, the findings from this assessment are foundational for scaling up GMP in EPI outreach sites (9).

Though better than the control upazilas, there were challenges in complying to the GMP protocol including quality of anthropometric measurements, counseling, and record keeping and reporting. Supervisory support needs to be enhanced, and additional trainings provided to improve quality of anthropometry and counseling. Digitalization of GMP data and analytics could reduce errors in reporting on children affected by growth faltering but needs to be tested. Furthermore, through the EPI platform, children up to age two were reached, and not according to the recommend frequency of weight and height measurement at every month and every 3 months, respectively. Thus, in addition to increasing the availability of GMP services at EPI sites, the use of community clinics needs to be promoted to ensure growth tracking as per recommendation, and counseling for all children under five.

Second, maternal nutrition services of weight measurement, screening for anemia, and provision of IFA and calcium supplements had better coverage in intervention upazilas. However, there were missed opportunities in providing comprehensive and continuing ANC services, counseling on diets, and consumption of IFA and calcium supplements. Home visits are critical platform for individual counseling on maternal nutrition. Among recently delivered women who deliver at home, the need for home visits is enhanced as women have restricted mobility the first few weeks after delivery due to health and/or cultural reasons. The coverage of home visits did not match the expected 15 to 30% reported home deliveries in Begumganj and Kaliganj, respectively (as per recent monitoring data). Under community-based engagement, the attempt was to regularize these visits with additional job aids and closer monitoring of these visits through supervisors. However, the assessment revealed that CHWs did not revisit a hamlet already covered in a month, despite births being reported from the area. The 24-h window was thus missed in most cases. The high proportion of adolescent pregnancies and young mothers in the survey exacerbated the issue of low domiciliary visits to this target group.

The augmented services did not have any impact on behavior change related to mothers’ diet and infant and young child feeding practices. This is contradictory to the findings from a recent study in an urban setting. Provision of nutritional services including counseling through dedicated staff at urban health centers increased dietary diversity among women and resulted in a 7% increase in early initiation of breastfeeding when compared to control areas (18). This difference between our study could be related to intervention elements including a dedicated nutrition cadre/staff, study setting, and duration of intervention. Quality of counseling needs concerted attention as counseling was reportedly being provided but not translating to behavior change. Group counseling through courtyard meetings requires a rethink in design and implementation.

Tools and training were provided to equip service providers and supervisors with the necessary skills and knowledge to deliver MIYCN services effectively. In addition, facilitatory materials, including job aids and protocols, were developed to provide clarity and guidance on roles. The supportive supervision component improved as CHWs nutrition roles were included in the scope of the supervision process and reporting. However, challenges in accurate and complete reporting continued despite simplification of supervision checklists. The implementation of the strategy required coordinated efforts at the national, district, and upazila levels. High-level oversight was provided by relevant authorities to monitor progress and inform planning and budget allocation. Meeting the pre-requisites to implement the service package and continual technical assistance at national, district, and upazila levels were possible through UNICEF and FHI360 support. Improving the visibility of nutrition services at the upazila level, so that they are integrated with EPI and family planning services, requires a coordinator to oversee nutrition programming, as was done in the 12 implementation upazilas.

Innovations led by Union Parishads in selected areas such as provision of SBC materials and transport support for volunteers have application in other similar contexts but require coordination between department of local government and upazila managers from health and family planning.

The study identified several systemic issues that could impact the effectiveness of MIYCN services. These included challenges related to human resource gaps, inadequate training, and coordination issues between relevant authorities. Additional capacity building is required in the form of refresher training or capsule training during monthly meetings. In future, additional training days should be considered in the community-based engagement cascade training program. There were also concerns about the sustainability of certain interventions, such as joint DGFP and DGHS review meetings, post-project completion.

## 5 Limitations

One major limitation of the quantitative study was the single time-point data collection design. While the assessment provided valuable insights into the current state of community-based engagement implementation, the absence of baseline measurements made it difficult to determine whether the observed outcomes were solely a result of the intervention or were influenced by external factors. The study, therefore, was unable to conclusively determine whether the observed values at the endline represent an improvement, decline, or simply the existing status quo prior to the intervention. Furthermore, the differences in background characteristics between intervention and control upazilas may have influenced some findings, particularly those impacted by belonging to different wealth index categories such as dietary diversity. As the strategy was implemented in the most challenging upazilas in each district, comparing them with other upazilas has limitations. Some of the gains in dietary diversity and child feeding practices may be masked in the intervention upazilas due to this. Furthermore, the control upazila for Begumganj received multi-year donor investment to strengthen maternal and newborn care services which may have resulted in better performance on maternal nutrition indicators. Disaggregating data by upazilas was crucial for assessing implementation in areas with varying governance and previous donor investments, while the study was designed to compare overall performance between the intervention and control upazilas. A combined analysis was conducted on a limited set of data points, but no significant differences were found when compared to the disaggregated analysis. In the household survey, while service utilization data were verified through records, responses on behaviors-related questions specifically on maternal diet and infant and child feeding were susceptible to recall bias. Limitations were also noted in planning observation sessions. Providing prior notification may have influenced CHW preparation and organization of the sessions, potentially not presenting the typical service delivery scenario. Efforts were made to minimize any obtrusive effects, but the possibility that CHWs modified their normal practices due to the scheduled observations cannot be ruled out.

## 6 Conclusion

The community-based engagement strategy was successful in increasing availability of child weight and MUAC measurement, counseling, and referral for suspected growth faltering in EPI outreach sites. Compliance to recommended GMP service protocol was also better in intervention upazilas although more time is needed to improve screening and counseling as GMP in outreach sites is a new service for CHWs. These services have the potential to scale, provided pre-requisites of equipment, protocol, training, volunteer support in high EPI case load sites, supervision, and leadership support are in place.

Among maternal nutrition services, introduction of maternal nutrition protocol for ANC along with CHW training and supervision improved gestational weight gain monitoring, height measurement, and provision of IFA and calcium supplements. Finally, replacement of multiple health and nutrition records with a single mother and child health and nutrition card was feasible and received high acceptance by all stakeholders.

At upazila level, integrating GMP, satellite clinics, and courtyard meetings into EPI microplans was a crucial management strategy to ensure ownership by upazila managers. Continued investments are needed in capacity building of service providers and supervisors especially on SBC, equipping service delivery points and CHWs, maintaining access to protocols and SBC resources, institutionalizing annual microplanning, progress review, and supervisory visits inclusive of MIYCN services. Dedicated nutrition cadre at upazila level is essential for technical oversight and prioritizing nutrition services. Concomitantly, systemic challenges of vacancies and intra-and inter-ministerial coordination need to be addressed to ensure the sustainability and scalability of interventions.

## Data Availability

The raw data supporting the conclusions of this article will be made available by the authors, without undue reservation.
